# Chronic Cough in Musculoskeletal disorders: Using high resolution oesophageal manometry in search of an Aetiology

**DOI:** 10.1186/1745-9974-8-6

**Published:** 2012-09-28

**Authors:** Sega Pathmanathan, Jaymin B Morjaria, Warren Jackson, Alyn H Morice

**Affiliations:** 1 Division of Cardiovascular and Respiratory Studies, Hull York Medical School, University of Hull, Castle Hill Hospital, Cottingham HU16 5JQ, UK; 2 Department of Gastro-Intestinal Physiology, Castle Hill Hospital, Cottingham UK

**Keywords:** Chronic cough, Airway reflux, Ehlers-Danlos, Dermatomyositis

## Abstract

Chronic cough is a common symptom carrying significant morbidity which can occur as a result of oesophageal dysmotility. Here we report 2 patients with musculoskeletal disease presenting with chronic cough to our tertiary cough clinic. Prior to referral both patients had been extensively investigated to determine the basis of their cough, with no cause found. Oesophageal studies, using high resolution oesophageal manometry, demonstrated oesophageal dysmotility with consequent airway reflux. Anti-reflux therapy resulted in a good response in both patients. These are the first reports of the recently developed technique of high resolution manometry aiding the diagnosis of chronic cough. This technique may provide important clues into aetiological mechanism in patients with conditions predisposing to reflux into the airways.

## Background

Chronic cough is one of the most commonly reported medical symptom with 12% of the population reporting symptoms daily or weekly [1]. It may be associated with significant morbidity. The differential diagnosis of chronic cough is extensive and rare causes may be overlooked without a perceptive history and examination coupled to appropriate investigations. Reports suggest that up to two thirds of patients with chronic cough have oesophageal dysmotility [2]. Here we describe two patients with chronic cough and musculo-skeletal disease from our tertiary cough clinic in whom the clinical suspicion of oesophageal dysmotility was confirmed by high resolution manometry.

### Case 1

A 52-year old woman presented with a 2-year history of cough, often exacerbated by singing. This was associated with a hoarse voice and intermittent wheeziness. She was an ex-smoker, having stopped 18 months previously, with less than 10-pack years of smoking.

She had a background medical history of dermatomyositis diagnosed in 2008 managed with methotrexate. Eight months following her initial diagnosis, she had developed heartburn. At her local clinic, her chest x-ray, routine blood tests, and spirometry were within the normal range. For her cough she was initiated on inhaled, budesonide and salbutamol, with no benefit.

**Figure 1:**
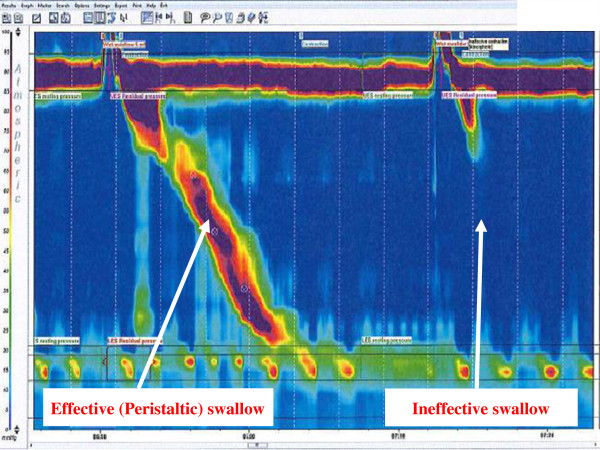
High resolution manometry plots of an effective and ineffective swallow in the patient with dermatomyositis.

Physical examination was unremarkable and she had a score of 13 on the Hull Airways Reflux Questionnaire (HARQ) (normal range <13) [3].We arranged for her to have a full oesophageal assessment as well as a cough challenge. High resolution oesophageal studies (Solar GI HRM, Medical Measurement Systems, Enchede, Netherlands) showed partial lower oesophageal sphincter (LOS) relaxation in co-ordination with the upper oesophageal sphincter (UOS), with only 50% of her wet swallows being effective (normal range >90%) (Figure 1). Her mean LOS was 26 mmHg (Normal 15-30 mmHg) while her UOS pressure was 129 mmHg (Normal 34–104 mmHg). A focal area of oesophageal dysmotility was noted at 20 cm from the nose.

She was commenced on metoclopramide 10mgs three times a day, and to-date, after 12 months of treatment, her cough remains abated.

### Case 2

A 56-year old lady was referred to our clinic with an 18 month history of cough, change in character of her voice and symptoms of post-nasal drip on rising from bed. There was an associated metallic taste in her mouth. She had had a long history of heartburn which was treated with a proton pump inhibitor (PPI). Although on medication her heartburn symptoms were controlled, the cough still persisted. She had a score of 38 on the HARQ.

Her past medical history comprised of Ehlers-Danlos syndrome, multiple TIA’s with no residual neurological deficit, well controlled sinus symptoms and a hysterectomy for fibroids. She had a family history of asthma. Initial investigations, at her local hospital revealed normal blood tests, chest x-ray, beta agonist reversibility testing and oral corticosteroid challenge. Despite this she was continued on salmeterol/fluticasone and salbutamol inhalers with no benefit. She also took aspirin 150 mg daily, pantoprazole 20 mg daily, and co-codamol as required.

**Figure 2:**
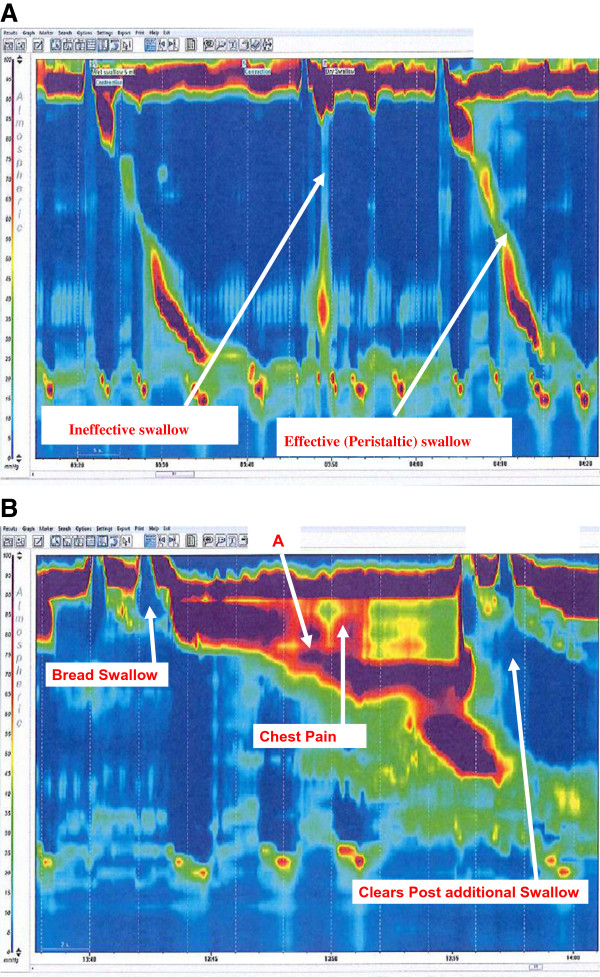
**High resolution manometry plots of an effective and ineffective swallow in the patient with Ehlers-Danlos syndrome. **This patient only had 50% effective swallows. High resolution plot of significant oesophageal spasm at 11 cm resulting in bolus arrest (marked **A** on the figure) in the patient with Ehlers-Danlos syndrome.

High resolution oesophageal manometry demonstrated an adequate LOS pressure (22 mmHg) which relaxed in co-ordination with UOS (Figure 2A). The UOS pressure was 182 mmHg. Normal peristaltic contractions were noted with 80% of the wet swallows being effective. Of note, she experienced chest discomfort on solid bolus swallowing that correlated with an area of prolonged high pressure (bolus arrest with subsequent upper oesophageal spasm (Figure 2B)).

She was commenced on full dose acid suppression (pantoprazole 40 mg twice daily and ranitidine 300 mg at night) and baclofen. Akin to the first patient, her symptoms were well controlled at a year’s review.

## Discussion

Here we report two patients with chronic cough associated with conditions affecting the musculo-skeletal system. Both had oesophageal studies which demonstrated oesophageal dysfunction as the probable cause.

The lower two thirds of the oesophagus is made up of smooth muscle, whilst the upper one third of striated muscle. Oesophageal complications are frequently found in musculoskeletal illness. In inflammatory myopathies, like dermatomyositis, this occurs in 8-30% of patients and can be the presenting complaint in a fifth of these [4,5]. Abnormalities in oesophageal motility are frequent and may involve both the upper and lower oesophagus [6]. The phenotypic dysmotility seen is similar to that seen in scleroderma; however the mechanism of dysfunction in scleroderma is thought to be due to the oesophageal smooth muscle being replaced by fibrous tissue. This has not been reported in dermatomyositis. The degree of involvement predicts the duration of symptoms [6]. It has also been reported that there may be a degeneration of the striated muscle supporting the LOS with decrease in the LOS pressure [7]. This can lead to reflux of both acidic and non-acid material into the oesophagus.

Oesophageal disorders are also recognized in Ehlers-Danlos syndrome, especially oesophageal rupture due to mega-oesophagus. However, whilst mega-oesophagus may result in airway reflux [8], an alternative mechanism could be of an altered physico-mechanical property of the oesophagus.

Oesophageal manometry is used to evaluate intraluminal pressure and muscular coordination in the oesophagus. It has been shown to have utility in diagnosing motility disorders such as achalasia, diffuse oesophageal spasm and scleroderma [9]. It is also frequently used to assess the degree of oesophageal motility before anti-reflux surgery. High resolution manometry (HRM) is a recent development that can detect localized abnormalities of oesophageal function and predict bolus transport more accurately than conventional manometry [10]. At our centre we use a catheter with 36 sensors 1 cm apart. The sensors depict a spatiotemporal plot of the direction and force of pressure activity in the oesophagus from the pharynx to the stomach. Each pressure is assigned a colour. Instead of displaying multiple line plots, a single coloured plot is reported which is much easier to appreciate (see Figures 1 and 2). Hence, the physiology of oesophageal function is clearly demonstrated including the simultaneous relaxation of the upper oesophageal sphincter and lower oesophageal sphincter, the pressure trough between the proximal (striated muscle) and distal (smooth muscle) tubular oesophagus and the increasing pressure and duration of the peristaltic wave as it passes distally. This method of assessing patients provides a more detailed profile of the oesophageal function and displays plots that may be interpreted rapidly.

These are the first reported cases in the literature demonstrating the ability of high resolution manometry to identify subtle abnormalities of oesophageal function in the diagnosis of airway reflux-induced cough in musculoskeletal disease. Not only do these cases highlight the importance of a need for a high degree of clinical suspicion in cough associated with other conditions, but also reveal how new techniques of investigation offer insights into the role of oesophageal dysmotility in the aetiology of cough.

### Consent

Written informed consent was obtained from the patients for publication of this case report and copies of the consent may be made available to the editor-in-chief for viewing.

## Abbreviations

GORD: Gastro-oesophageal reflux disease; LOS: Lower oesophageal sphincter; UOS: Upper oesophageal sphincter; TIA: Transient ischaemic attack; PPI: Proton-pump inhibitor.

## Competing interests

No conflict of interest to declare for any of the authors.

## Authors’ contributions

SP and JBM have contributed equally to writing up the case report. The patients were seen by AHM. WJ conducted and interpreted the high resolution manometry. SP and JBM drafted the initial manuscript, and “all authors have read and approved the final manuscript for publication.”

## Declarations

JBM has received honoraria for speaking and financial support to attend meetings from Chiesi, Pfizer, MSD, Boehringer Ingelheim and GSK/Allen & Hanburys. AHM has received honoraria for speaker meetings and financial support to attend meetings/advisory boards from Chiesi, Pfizer, MSD, Boehringer Ingelheim, Novartis, GSK, AstraZeneca, Proctor & Gamble Healthcare, Orion Respiratory UK, Vectura Ltd, and Nycomed.
